# Attachment styles and empathy in trainee nurses: the mediating and moderating roles of attitudes toward death

**DOI:** 10.3389/fpsyg.2024.1445587

**Published:** 2024-08-05

**Authors:** Ting Wei, Meiyi Guo, Huanle Jin, Bingren Zhang

**Affiliations:** ^1^School of Nursing, Hangzhou Normal University, Hangzhou, China; ^2^Affiliated Hospital (School of Clinical Medicine), Hangzhou Normal University, Hangzhou, China

**Keywords:** attachment style, attitude toward death, empathy, mediating and moderating effects, trainee nurse

## Abstract

**Aim:**

A growing body of evidence has shown that attachment styles and death attitudes have a significant impact on empathy. This study aimed to explore the precise role of death attitudes in the relationship between attachment styles and empathy levels among trainee nurses.

**Methods:**

A total of 626 Chinese trainee nurses with different attachment types were enrolled, and their attachment styles, death attitudes, and empathy levels were assessed using the Revised Adult Attachment Scale, the Death Attitude Profile-Revised, and the Jefferson Scale of Empathy-Healthy Professionals, and finally, data from 566 participants were included for statistical analysis.

**Results:**

We found that among trainee nurses with secure attachment type, fear of death, approach acceptance, escape acceptance, and neutral acceptance (−) mediated the relationships between attachment-related avoidance/anxiety and their overall empathy levels and all its dimensions; in the preoccupied type, only neutral acceptance (−) mediated the relationships between attachment-related avoidance and their overall empathy levels and compassionate care; and in the fearful type, only fear of death mediated the relationship between attachment-related avoidance and compassionate care. Furthermore, in the secure type, neutral acceptance attenuated the negative predictions of attachment-related avoidance on overall empathy level and perspective taking.

**Conclusion:**

Attitudes toward death played different mediating and moderating roles in the relationship between attachment styles and empathy among trainee nurses with different attachment types. In addition to acculturated empathy-specific training, targeted education related to death for trainee nurses with different attachment types is needed to prevent their compassion fatigue.

## 1 Background

Empathy is an emotional (affective) response that depends on the interplay of trait abilities and state influences and is analogous to a person’s perception (reflecting emotional empathy) and understanding (reflecting cognitive empathy) of another’s emotions (Cuff et al., 2016). In clinical settings, empathy includes the ability to recognize and temporarily experience a patient’s emotional state from visual and verbal cues (Hirsch, 2007; Decety, 2020), to understand the patient’s situation, thoughts, and feelings, and to feed this understanding back to the patient (Coulehan et al., 2001; Harrison, 2021), which can improve the quality of care and together with the absence of prejudice promotes a therapeutic nurse–patient relationship (McKenna et al., 2012; Decety, 2020). However, an increased number of studies reported that nurses worldwide were suffering from symptoms of compassion fatigue, a state of loss or lack of empathy, particularly emotional empathy, which is often accompanied by burnout (Decety, 2020; Graves et al., 2023). As early as 2005, Walker and Avant (2005) noted that prolonged self-sacrifice and/or prolonged exposure to adversity can lead to compassion fatigue. More recently, a meta-narrative review by Sinclair et al. (2017) suggested that compassion fatigue should be critically revisited as a work-related stress response for healthcare providers, including nurses. Recent investigations and systematic reviews showed a dramatic increase in the incidence of such phenomenon among nurses, especially after the COVID-19 pandemic (Amir and Okalo, 2022; Garnett et al., 2023), which triggered an increase in burnout and even turnover among nurses in several departments such as emergency department, hemodialysis unit, and oncology (Wells-English, 2019). Literature indicated that objective factors, such as the COVID-19 pandemic, high workload, extensive working hours, and unsupportive work environment, as well as young age, gender being women, and low education level of practitioners, contributed to such a lowered level of empathy among nurses (e.g., Garnett et al., 2023; Hu et al., 2023). However, even without these objective factors, some nursing practitioners were still vulnerable to compassion fatigue. For example, compassion fatigue was reported to lead to decreased wellbeing, program withdrawal, and intention to leave nursing among undergraduate, pre-licensure nursing students (Chachula, 2022). Before the COVID-19 pandemic, a study among nursing students showed that even contact with patients for not long periods of time could reduce their empathy (Ward et al., 2012). In addition to the possible effects of younger age and incomplete nurse education, as some studies reported (Garnett et al., 2023; Hu et al., 2023), intrinsic factors may also be underlying causes, such as personality (Melchers et al., 2016), self-efficacy, coping skills (Chachula, 2022), the tendency to explicitly adopt others’ perspectives in addition to understanding them when cognitive empathy is involved (Decety, 2020), and strong emotional and physiological reactivity after perception of other’s feelings in emotional empathy (Pérez-Chacón et al., 2021).

## 2 Introduction

Attachment style is the pattern of emotional connection with a primary caregiver (usually a parent) that develops as an individual grows up and influences the way an individual builds relationships with others in adulthood (Hinde, 1969). Specific combinations of attachment styles give rise to the formation of attachment types (Collins, 1996). Indeed, research has shown that attachment style can influence emotional and cognitive empathy in young adults through self-emotional regulation (Henschel et al., 2020). In addition, new parents with more secure and less anxious-ambivalent and avoidant attachment manifestations (namely the secure type) were better able to perceive and express empathy in interpersonal relationships, and they are generally more pleasant to parents (Kazmierczak, 2015). In contrast, individuals with an anxious-ambivalent attachment style developed an unstable attachment type in adulthood (namely the preoccupied type), which led them to show high levels of empathy at times and the opposite at others (De Sanctis and Mesurado, 2022), with interpersonal emotion regulation efficacy and self-concept as moderator and mediator, respectively, in such cases (George-Levi et al., 2022; Kural and Kovács, 2022). Among nursing students, attachment style was also found to be a predictor of empathy levels, with secure attachment being positively correlated with empathy and insecure attachment, such as avoidant and anxiety styles, the opposite (Khodabakhsh, 2012).

Meanwhile, attitudes toward death reflect one’s feelings about the overall concept of death, whether it is one’s own death or the death of others (Wong et al., 1994). A positive attitude toward death can be defined as a rational view of death as a natural phenomenon, which enables individuals to cope effectively with the negative emotions associated with death, while a negative attitude toward death is manifested by a fear of death, avoidance of death, or a perception of death as a means of escaping from reality (Wong et al., 1994; Peng et al., 2018). Evidence indicated that attachment style had an impact on attitudes toward death. For instance, insecure attachment style has been widely accepted as a risk factor for complicated grief after bereavement (e.g., Huh et al., 2017; Russ et al., 2022), and it may increase the risk of suicidal thoughts and behaviors in individuals (Green et al., 2020), revealing a tendency of using death as a way to escape from real problems among these individuals. A study on nurses of different departments also indicated that those with dependent rather than anxious-ambivalent attachment styles had more positive attitudes toward death, manifested by less fear of death (Gama et al., 2012). On the other hand, some scholars proposed that insecure attachment had a broader adaptive function according to the social defense theory, and different attachment orientations, including both the secure and insecure ones, could work synergistically to promote group survival (Ein-Dor and Hirschberger, 2016). In general, the above evidence implied that insecurely attached individuals had a much more mixed picture of the impact on their attitudes toward death than securely attached ones.

Interestingly, evidence also suggested that attitude toward death had a potential influence on empathy. A systematic review reported that associations between participation in assisted dying services and mental health outcomes, such as anxiety and mental distress, have not been consistently observed and that a potential influencing factor is the perceived competence of the patient’s care (Wibisono et al., 2022). Healthcare students and staff who were more anxious about death-related events tended to alleviate this anxiety by empathizing with those who were not dying (Servaty et al., 1996). Such findings seemed to support a positive correlation between higher levels of death anxiety and empathy among individuals majoring in nursing. While more recently, Medina-Fernández et al. (2023) found that among nurses in the intensive care unit, fear of and coping with death exacerbated compassion fatigue, Han et al. (2023) also reported that among undergraduate nursing students, the ability to empathize with others was positively correlated with positive death attitudes and negatively correlated with negative death attitudes. Combined with the diverse expressions of empathy among individuals with different attachment styles (Khodabakhsh, 2012; Kazmierczak, 2015; De Sanctis and Mesurado, 2022), we inferred that, in addition to the department in which they were located (Garnett et al., 2023), the discrepancy between the evidence might also be correlated with the selection of subjects with different attachment types, that is, unlike subjects with secure attachment types, those with insecure attachment types could hold more negative attitudes toward death, and such attitudes might further reduce their empathy levels in the clinical setting.

It is also plausible that attitude toward death played a moderating role between attachment styles and empathy among nurses. Supportive evidence demonstrated that education about the meaning of life broadened the understanding of death among medical students, thereby increasing their level of empathy (Damiano et al., 2017). Similarly, pharmacy students who participated in a 5-week elective course on death and dying experienced positive changes in fear of death, and their empathy levels were improved after completing the course (Manolakis et al., 2011). This change in attitude toward death may further enhance empathy levels of securely attached nurses and provide vital support to preoccupied nurses who exhibit dual empathy due to attachment instability (De Sanctis and Mesurado, 2022).

To sum up, attachment style is associated with attitudes toward death and empathy, but more research is needed to fully understand the pathways from specific attachment styles to empathy and their moderators among trainee nurses. This could enrich the theories related to the impact of attachment styles and attitudes toward death, and the factors influencing empathy on the one hand, and provide insights into the development of effective and targeted empathy training programs for nurses in the early stages of their careers on the other.

## 3 The study

Therefore, in this study, we aimed to clarify the exact relationships between attachment styles, attitudes toward death, and empathy among trainee nurses, in particular the role that attitudes toward death played in the relationships between attachment styles and empathy among those with different attachment types. We hypothesized that (1) trainee nurses with insecure attachment types generally had more negative attitudes toward death and lower empathy levels than those with secure ones; (2) among those with insecure attachment types, anxious and avoidant attachment styles were associated with more negative attitudes toward death and lower empathy levels; (3) insecure attachment styles contributed to lower empathy levels among trainee nurses through the mediation of negative attitudes toward death; and (4) attitudes toward death moderated the relationships between attachment styles and empathy.

## 4 Methods

### 4.1 Participants

A total of 626 trainee nurses from a leading hospital in Hangzhou were invited to participate in the survey. These nurses were self-reported to have no current acute or chronic illness and had no history of psychiatric or neurological abnormalities. All the participants were Han Chinese, had been employed as trainee nurses in the hospital for less than 6 months, and rotated between different departments such as medicine, surgery, gynecology, and pediatrics. A total of 60 participants were excluded due to unclear attachment type as they fell on the boundary of more than one attachment type. Finally, 566 participants were enrolled, and their sociodemographic characteristics and empathy levels are shown in Table 1. The study was approved by the Ethics Committee of Hangzhou Normal University School of Nursing, and all the participants volunteered to participate in the study.

**Table 1:** Empathy levels (measured by the Jefferson Scale of Empathy-Health Professionals, JSE-HP) in trainee nurses with different sociodemographic characteristics (*N* = 566).

Variables	*n* (%)	JSE-HP total score	*t*/*F*	*p*
Gender			0.02	0.98
Men	51 (9.0)	117.47 ± 12.65		
Women	515 (91.0)	117.43 ± 12.82		
Age (years)			1.62	0.11
19 ~ 22	452 (79.9)	117.87 ± 12.80		
23 ~ 26	114 (20.1)	115.71 ± 12.66		
Education level			0.74	0.53
Junior college	53 (9.4)	119.66 ± 14.35		
Undergraduate student	495 (87.5)	117.24 ± 12.48		
Master candidate	18 (3.2)	116.17 ± 16.22		
Attachment type			10.07	<0.001
Secure type	396 (70.0)	119.13 ± 12.11		
Preoccupied type	101 (17.8)	115.12 ± 12.80*		
Dismissing type	15 (2.7)	115.07 ± 14.93		
Fearful type	54 (9.5)	110.04 ± 13.93***		

*p < 0.05 vs. the secure type; ***p < 0.001 vs. the secure type.

### 4.2 Measures

#### 4.2.1 The Revised Adult Attachment Scale (RAAS)

The RAAS is a scale that measures how an individual feels about close relationships (Collins, 1996). It consists of 18 questions answering on a 5-point Likert scale (1—not at all characteristic of me to 5—very characteristic of me). The scale contains three dimensions, namely, Close, Depend, and Anxiety, each consisting of six items. The Close dimension measures a person’s comfort level with closeness and intimacy, and the Depend dimension measures the extent to which a person feels he/she can rely on others in times of need. These two can be averaged and then reverse-scored to form an overall index of attachment-related avoidance. The Anxiety dimension measures the degree to which a person worries about being rejected or unloved, including attachment-related anxiety. Individuals with secure attachment type have low levels of both attachment-related avoidance and anxiety; individuals with preoccupied type have low levels of attachment-related avoidance but high levels of anxiety; individuals with fearful type have high levels of both attachment-related avoidance and anxiety; and individuals with dismissing type have low levels of attachment-related anxiety but high levels of avoidance. The Chinese version of this scale had good reliability and validity (Wu et al., 2004), and its Cronbach’s alpha in this study was 0.70.

#### 4.2.2 The Death Attitude Profile–Revised (DAP-R)

The DAP-R (Wong et al., 1994) is a scale to assess attitudes toward death and comprises 32 items distributed across five dimensions: fear of death (7 items) reflects negative thoughts and feelings about the state of death, death avoidance (5 items) reflects defense mechanism that keeps death away from one’s consciousness, neutral acceptance (5 items) reflects the view of death as a reality that is neither to be feared nor welcomed, approach acceptance (10 items) reflects the view of death as a gateway to a happy afterlife, and escape acceptance (5 items) reflects the view of death as an escape from a painful existence. Each item was scored on a 5-point Likert scale (1—strongly disagree to 5—strongly agree). The Chinese version of this scale was reliable and valid in a previous study (Tang et al., 2014), and its Cronbach’s alpha in this study was 0.90.

#### 4.2.3 The Jefferson Scale of Empathy-Healthy Professionals (JSE-HP)

The JSE-HP (Hojat et al., 2002) is a widely used scale for evaluating medical staff’s empathy level, which consists of 20 items rated on a 7-point Likert scale (10 of them are directly scored as 1—fully disagree to 7—fully agree, and the other 10 are reversely scored). The scale contains three dimensions, namely, perspective taking (ability to understand patients’ perspectives, i.e., cognitive empathy), compassionate care (expression of caring, concern, and empathy), and walking in the patient’s shoes (feeling what patients feel, i.e., emotional empathy). The higher the total or specific subscale score is, the more related empathy one has. The scale has been demonstrated to be reliable and valid in samples from different countries (Hojat et al., 2018), and its Cronbach’s alpha in this study was 0.88.

### 4.3 Statistical analyses

The statistical analyses were conducted using the IBM SPSS v26.0 software, with the addition of process plug-ins for moderation and mediation analyses. A descriptive analysis was used to describe the sociodemographic variables of the trainee nurses. The Student’s *t*-test and one-way ANOVA were used to assess the JSE-HP scores of participants with varying sociodemographic characteristics. Once group differences in the JSE-HP total score by gender, age, or education level were identified, these variables would be used as covariates in further analyses. Subgroup analyses would be conducted if group differences in the JSE-HP by attachment type were identified. A *p*-value of less than 0.05 was considered statistically significant, as were all subsequent *p*-values.

Hypothesis 1 was tested using a one-way ANOVA to compare the RAAS, DAP-R, and JSE-HP scale scores in trainee nurses with different attachment types. Effect sizes were calculated using η^2^. Once a main effect on scale score was identified, a *post-hoc* Bonferroni test was employed.

Hypothesis 2 was tested with Pearson’s correlation analysis to explore the relationships between attachment styles, attitudes toward death, and empathy levels among trainee nurses with different attachment types.

**Figure 1:**
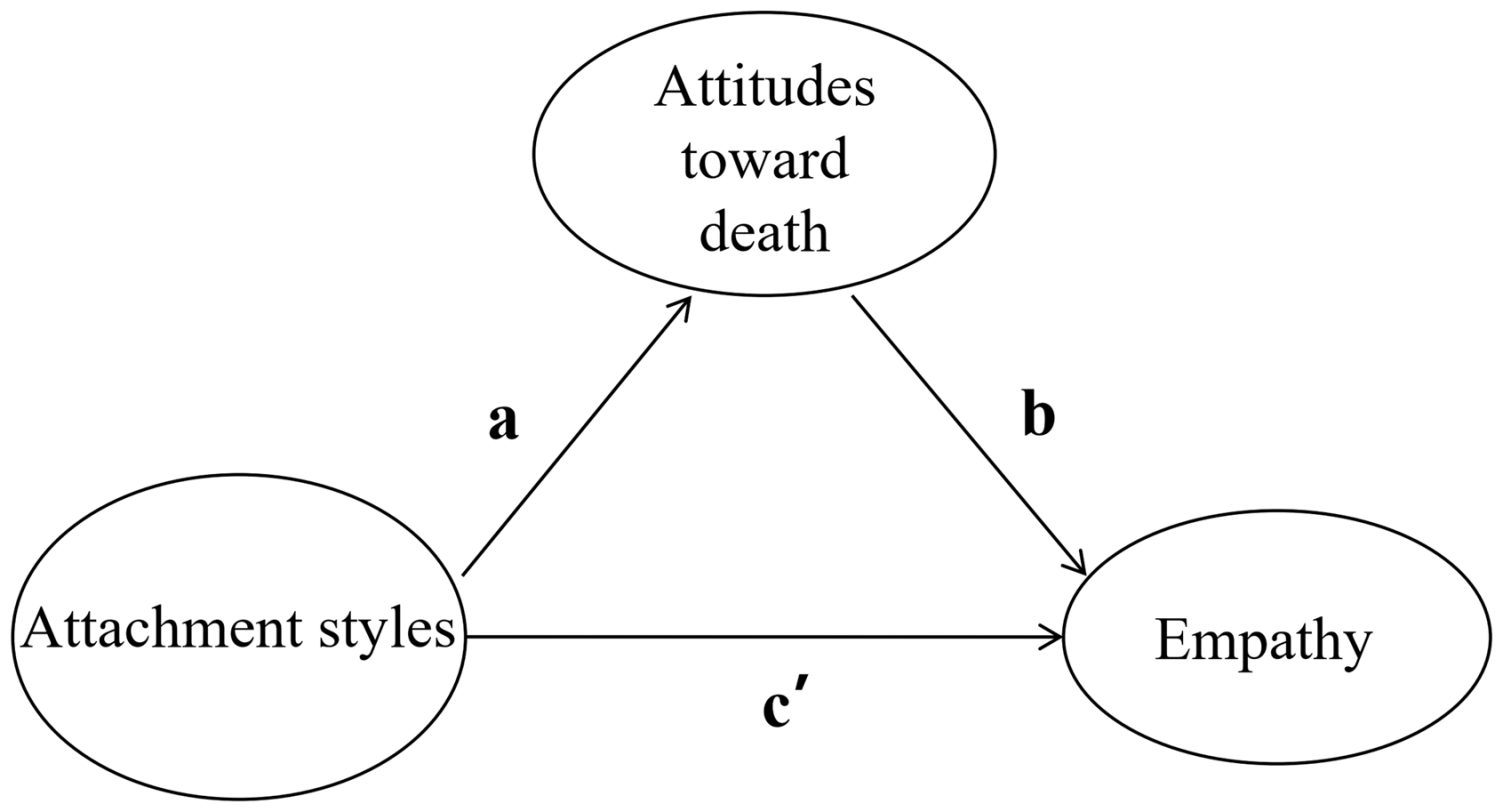
Theoretical mediation model in this study. The model consists of 160 sub-models based on the potential mediating role of the five attitudes toward death between the two attachment styles and overall empathy and its three dimensions among nurses of the four attachment types.

For hypotheses 3 and 4, least square regression (bootstrap method) was used to analyze the mediating and moderating roles of death attitudes in PROCESS (Hayes, 2017). Each of the two attachment styles was used as an independent variable, and empathy and its dimensions were used as dependent variables. For mediation analysis, as shown in Figure 1, the letters a, b, and c, respectively, represented path coefficients that reported the relationships from attachment styles to attitudes toward death, from attitudes toward death to empathy, and from attachment styles to empathy. To eliminate the multicollinearity effects between the variables, the data were centralized prior to analysis. The 95% confidence intervals (CIs) were estimated by the bias-corrected bootstrapping procedure, and the number of iterations was set to 5,000. A non-zero 95% CI for the interaction term and indirect effect indicated that specific attitudes toward death had significant mediating and moderating roles in the indirect effects and interaction terms.

## 5 Results

As shown in Table 1, no significant differences were found in JSE-HP total scores based on gender, age, or education level (*p*s > 0.05), while a significant difference was found among those with different attachment types (*F* [3, 562] = 10.07, mean square effect (MSE) = 156.1, *p* < 0.001, η^2^ = 20), with preoccupied type (*p* < 0.05) and fearful type (*p* < 0.001) scoring lower than those with secure type.

### 5.1 Group differences in attachment styles, death attitudes, and empathy among trainee nurses with different attachment types

As shown in Table 2, significant differences were found in scores of RAAS anxiety and avoidance (*F* [3, 562] = 289.44–464.07, MSE = 0.29–0.30, *p*s < 0.001, η^2^ = 0.61–0.71), DAP-R fear of death, death avoidance, escape acceptance, neutral acceptance, and approach acceptance (*F* [3, 562] = 4.29–19.58, MSE = 7.66–23.90, *p*s < 0.01, η^2^ = 0.02–0.10), as well as JSE-HP compassionate care, walking in patient’s shoes, and perspective taking (*F* [3, 562] = 4.14–12.11, MSE = 4.72–46.85, *p*s < 0.01, η^2^ = 0.02–0.06) among trainee nurses with different attachment types. *Post-hoc* test showed that concerning attachment style, compared to the secure type, the preoccupied (*p* < 0.001), fearful (*p* < 0.001), and dismissing types (*p* < 0.001) scored higher on RAAS avoidance, while preoccupied (*p* < 0.001) and fearful (*p* < 0.001) types scored higher on RAAS anxiety; compared to the preoccupied type, the fearful type scored higher on both RAAS avoidance (*p* < 0.001) and anxiety (*p* < 0.001), and the dismissing type was higher on RAAS avoidance (*p* < 0.001) while lower on anxiety (*p* < 0.01); and compared to the dismissing type, the fearful type was higher on RAAS anxiety (*p* < 0.001).

**Table 2:** Mean scores (±S.D.) of the Revised Adult Attachment Scale, Death Attitude Profile-Revised, and Jefferson Scale of Empathy-Health Professionals in trainee nurses with different attachment types (*N* = 566).

	Secure type (*n* = 396)	Preoccupied type (*n* = 101)	Dismissing type (*n* = 15)	Fearful type (*n* = 54)	*F*	MSE	*p*	η^2^
**Revised Adult Attachment Scale**								
Avoidance	1.63 ± 0.56	2.19 ± 0.54a,c	3.40 ± 0.35a,b	3.77 ± 0.47a,b	289.44	0.29	<0.001	0.61
Anxiety	1.83 ± 0.57	3.63 ± 0.46a,c	2.04 ± 0.51b	3.94 ± 0.47a,b,c	464.07	0.30	<0.001	0.71
**Death Attitude Profile-Revised**								
Fear of death	16.95 ± 4.86	20.58 ± 4.62a,c	16.20 ± 5.45b	20.17 ± 5.43a,c	19.58	23.90	<0.001	0.10
Death avoidance	13.21 ± 3.75	14.66 ± 3.50a	12.87 ± 4.94	14.72 ± 3.81a	5.97	14.04	0.001	0.03
Escape acceptance	11.10 ± 4.20	13.13 ± 3.92a	13.33 ± 6.10	14.63 ± 4.32a	4.29	17.80	<0.001	0.02
Neutral acceptance	20.73 ± 2.73	20.49 ± 2.36	21.40 ± 5.04	19.37 ± 2.93a	11.95	7.66	0.005	0.06
Approach acceptance	24.90 ± 6.71	28.05 ± 5.94a	26.47 ± 9.27	29.56 ± 6.71a,c	15.69	11.95	<0.001	0.08
**Jefferson Scale of Empathy-Health Professionals**								
Perspective taking	59.21 ± 6.82	57.38 ± 6.77	57.40 ± 7.93	56.41 ± 6.83a	4.14	46.85	0.006	0.02
Compassionate care	48.16 ± 5.34	46.43 ± 5.83a	46.33 ± 7.68	43.37 ± 7.94a,b	12.11	33.47	<0.001	0.06
Walking in patient’s shoes	11.75 ± 2.13	11.32 ± 2.19	11.33 ± 2.50	10.26 ± 2.37a,b	7.90	4.72	<0.001	0.04

a, *p* < 0.05 vs. the secure type; b, *p* < 0.05 vs. the preoccupied type; c, *p* < 0.05 vs. the dismissing type.

In terms of attitude toward death, compared to the secure type, the preoccupied and fearful types scored higher on DAP-R fear of death (*p*s < 0.001), death avoidance (*p*s < 0.05), escape acceptance (*p*s < 0.001), and approach acceptance (*p*s < 0.001), and the fearful type scored lower on neutral acceptance (*p* < 0.01); compared to the dismissing type, the preoccupied (*p* < 0.01) and fearful (*p* < 0.05) types scored higher on fear of death.

As to the empathy level, compared to the secure type, the preoccupied type scored lower on compassionate care (*p* < 0.05), the fearful type scored lower on perspective taking (*p* < 0.05), walking in patients’ shoes (*p* < 0.001), and compassionate care (*p* < 0.001) and compared to the preoccupied type, the fearful type scored lower on compassionate care (*p* < 0.05) and walking in patient’s shoes (*p* < 0.05).

### 5.2 Correlations between attachment styles, death attitudes, and empathy among trainee nurses with different attachment types

As presented in Table 3, in the secure type, RAAS avoidance and anxiety were positively correlated with DAP-R fear of death (*p*s < 0.01), death avoidance (*p*s < 0.05), approach acceptance (*p*s < 0.01), and escape acceptance (*p*s < 0.01) and negatively correlated with neutral acceptance (*p*s < 0.05), JSE-HP total score (*p*s < 0.01), and all its subscale scores (*p*s < 0.01). Except that no relationship was found between death avoidance and perspective taking (*p* > 0.05), their JSE-HP total score and all its subscales were positively correlated with neutral acceptance (*p*s < 0.01) and negatively correlated with fear of death (*p*s < 0.01), death avoidance (*p*s < 0.05), approach acceptance (*p*s < 0.01), and escape acceptance (*p*s < 0.01).

**Table 3:** Correlations between attachment styles (measured by the Revised Adult Attachment Scale, RAAS), attitudes toward death (the Death Attitude Profile-Revised, DAP-R), and empathy levels (the Jefferson Scale of Empathy-Health Professionals, JSE-HP-HP) in trainee nurses with different attachment types (*N* = 566).

	RAAS		DAP-R				
	Avoidance	Anxiety	Fear of death	Death avoidance	Neutral acceptance	Approach acceptance	Escape acceptance
**Secure type (*n* = 396)**							
Fear of death	0.21**	0.28^**^	–	–	–	–	–
Death avoidance	0.13*	0.18^**^	0.59^**^	–	–	–	–
Neutral acceptance	−0.13**	−0.12*	−0.30^**^	−0.13**	–	–	–
Approach acceptance	0.02^**^	0.36^**^	0.44^**^	0.32^**^	−0.09	–	–
Escape acceptance	0.25^**^	0.34^**^	0.34^**^	0.25^**^	−0.13*	0.72^**^	–
JSE-HP total score	−0.30^**^	−0.24^**^	−0.25^**^	−0.11*	0.38^**^	−0.27^**^	−0.41^**^
Perspective taking	−0.24^**^	−0.18^**^	−0.17**	−0.06	0.36^**^	−0.20**	−0.37^**^
Compassionate care	−0.27^**^	−0.23^**^	−0.26^**^	−0.12^*^	0.32^**^	−0.26^**^	−0.35^**^
Walk in patient’s shoes	−0.21^**^	−0.21^**^	−0.25^**^	−0.12^*^	0.20^**^	−0.24^**^	−0.29^**^
**Preoccupied type (*n* = 101)**							
Fear of death	0.03	0.26**	–	–	–	–	–
Death avoidance	0.01	0.04	0.57^**^	–	–	–	–
Neutral acceptance	−0.24*	−0.03	−0.21*	−0.18	–	–	–
Approach acceptance	0.05	−0.05	0.29^**^	0.22*	−0.09	–	–
Escape acceptance	0.13	0.07	0.12	0.06	−0.26^**^	0.42^**^	–
JSE-HP total score	−0.23*	−0.02	−0.11	−0.03	0.31**	0.02	−0.29**
Perspective taking	−0.11	−0.05	−0.11	0.001	0.32**	0.06	−0.19
Compassionate care	−0.31**	−0.01	−0.05	−0.04	0.27**	−0.01	−0.36^**^
Walk in patient’s shoes	−0.16	0.04	−0.14	−0.04	0.10	−0.16	−0.12
**Dismissing type (*n* = 15)**							
Fear of death	−0.03	0.16	–	–	–	–	–
Death avoidance	0.14	0.60*	0.49	–	–	–	–
Neutral acceptance	−0.35	0.19	0.26	0.62^*^	–	–	–
Approach acceptance	−0.10	0.31	0.47	0.43	0.49	–	–
Escape acceptance	0.05	0.42	0.12	0.48*	0.40	0.76**	–
JSE-HP total score	0.22	0.03	0.28	0.52*	0.60*	0.13	0.09
Perspective taking	0.31	0.18	0.26	0.47	0.43	−0.01	−0.10
Compassionate care	0.10	−0.02	0.21	0.56*	0.69**	0.20	0.26
Walk in patient’s shoes	0.03	−0.34	0.21	−0.12	0.10	0.18	0.06
**Fearful type (*n* = 54)**							
Fear of death	0.38**	0.02	–	–	–	–	–
Death avoidance	0.13	−0.01	0.52*	–	–	–	–
Neutral acceptance	0.19	0.17	−0.11	0.02	–	–	–
Approach acceptance	0.27	0.06	0.46**	0.60**	0.12	–	–
Escape acceptance	0.14	0.12	0.18	0.36**	0.23	0.74**	–
JSE-HP total score	−0.12	−0.06	−0.32**	−0.38**	0.35**	−0.42**	−0.38**
Perspective taking	0.10	0.12	−0.06	−0.05	0.37**	−0.06	−0.12
Compassionate care	−0.28*	−0.16	−0.45**	−0.55**	0.22	−0.59**	−0.48**
Walk in patient’s shoes	−0.04	−0.16	−0.20	−0.24	0.26	−0.33*	−0.29*

*p < 0.05; **p < 0.01.

In the preoccupied type, RAAS avoidance was negatively correlated with neutral acceptance (*p* < 0.05), JSE-HP total score (*p* < 0.05), and compassionate care (*p* < 0.01); RAAS anxiety was positively correlated with fear of death (*p* < 0.01). The JSE-HP total score and compassionate care were positively correlated with neutral acceptance (*p*s < 0.01) and negatively correlated with escape acceptance (*p*s < 0.01); perspective taking positively correlated with neutral acceptance (*p* < 0.01).

In the dismissing type, RAAS anxiety was positively correlated with death avoidance (*p* < 0.05). JSE-HP total score and compassionate care were positively correlated with death avoidance (*p*s < 0.05) and neutral acceptance (*p*s < 0.05).

In the fearful type, RAAS avoidance was positively correlated with fear of death (*p* < 0.01) and negatively correlated with compassionate care (*p* < 0.05). JSE-HP total score was positively correlated with neutral acceptance (*p* < 0.01) and negatively correlated with fear of death (*p* < 0.01), death avoidance (*p* < 0.01), approach acceptance (*p* < 0.01), and escape acceptance (*p* < 0.01); compassionate care was negatively correlated with fear of death (*p* < 0.01), death avoidance (*p* < 0.01), approach acceptance (*p* < 0.01), and escape acceptance (*p* < 0.01); perspective taking was positively correlated with neutral acceptance (*p* < 0.01); and walking in patient’s shoes was negatively correlated with approach acceptance (*p* < 0.05) and escape acceptance (*p* < 0.05).

### 5.3 Mediating role of death attitudes on the associations between attachment styles and empathy among trainee nurses with different attachment types

As shown in Table 4, in the secure type, DAP-R fear of death (all *p*s < 0.05), approach acceptance (all *p*s < 0.001), escape acceptance (all *p*s < 0.001), and neutral acceptance (−) (all *p*s < 0.05) acted as partial mediators in the associations of RAAS avoidance/anxiety with JSE-HP total score, as well as with all its subscales scores, except for the fully mediating role of escape acceptance in the association of RAAS anxiety with perspective taking (*p*s < 0.001).

**Table 4:** Mediating role of attitudes toward death on the associations between attachment styles and empathy levels in trainee nurses with different attachment types (*N* = 566).

Intermediary variable	Regression coefficient			Indirect effect	95%CI	Effect proportion
	a	b	c’			
**Secure type (*n* = 396): RAAS avoidance**						
**JSE-HP total score**						
Fear of death	1.83***	−0.50***	−5.53***	−0.92	−1.64, −0.42	14.23%
Neutral acceptance	−0.65**	1.52***	−5.47***	−0.98	−1.95, −0.21	15.23%
Approach acceptance	2.47***	−0.40***	−5.46***	−0.99	−1.78, −0.47	15.37%
Escape acceptance	1.87***	−1.04***	−4.50***	−1.95	−2.92, −1.16	30.22%
**Perspective taking**						
Fear of death	1.83***	−0.17*	−2.68***	−0.31	−0.69, −0.08	10.49%
Neutral acceptance	−0.65**	0.83***	−2.46***	−0.54	−1.07, −0.11	17.88%
Approach acceptance	2.47***	−0.16**	−2.59***	−0.41	−0.78, −0.16	13.53%
Escape acceptance	1.87***	−0.53***	−2.00***	−1.00	−1.52, −0.58	33.36%
**Walk in patients’ shoes**						
Fear of death	1.83***	−0.09***	−0.64**	−0.17	−0.30, −0.08	21.12%
Neutral acceptance	−0.65**	0.14***	−0.73***	−0.09	−0.21, −0.02	10.74%
Approach acceptance	2.47***	−0.07***	−0.65**	−0.16	−0.30, −0.07	20.05%
Escape acceptance	1.87***	−0.13***	−0.58**	−0.24	−0.40, −0.13	29.57%
**Compassionate care**						
Fear of death	1.83***	−0.24***	−2.20***	−0.43	−0.76, −0.19	16.35%
Neutral acceptance	−0.65**	0.56***	−2.28***	−0.36	−0.74, −0.08	13.61%
Approach acceptance	2.47***	−0.17***	−2.21***	−0.42	−0.76, −0.19	16.02%
Escape acceptance	1.87***	−0.38***	−1.93***	−0.71	−1.10, −0.41	26.83%
**Secure type (*n* = 396): RAAS anxiety**						
**JSE-HP total score**						
Fear of death	2.40***	−0.51***	−3.82***	−1.21	−2.10, −0.59	24.12%
Neutral acceptance	−0.55*	1.57***	−4.17***	−0.86	−1.86, −0.07	17.07%
Approach acceptance	4.18***	−0.39***	−3.40**	−1.63	−2.67, −0.85	32.40%
Escape acceptance	2.17***	−1.08***	−2.69**	−2.34	−3.41, −1.52	46.56%
**Perspective taking**						
Fear of death	2.40***	−0.18*	−1.71**	−0.43	−0.85, −0.11	20.08%
Neutral acceptance	−0.55*	0.85***	−1.67***	−0.47	−1.02, −0.04	21.95%
Approach acceptance	4.18***	−0.16**	−1.45**	−0.68	−1.22, −0.24	32.05%
Escape acceptance	2.17***	−0.56***	−0.92	−1.22	−1.78, −0.78	–
**Walk in the patient’s shoes**						
Fear of death	2.40***	−0.09***	−0.54**	−0.22	−0.37, −0.11	28.83%
Neutral acceptance	−0.55*	0.14***	−0.69***	−0.08	−0.20, −0.01	9.99%
Approach acceptance	4.18***	−0.06***	−0.50**	−0.26	−0.45, −0.11	34.05%
Escape acceptance	2.17***	−0.13***	−0.48**	−0.28	−0.45, −0.15	36.6%
**Compassionate care**						
Fear of death	2.40***	−0.24***	−1.57***	−0.57	−0.97, −0.27	26.49%
Neutral acceptance	−0.55*	0.57***	−1.82***	−0.31	−0.13, −0.004	14.74%
Approach acceptance	4.18***	−0.16***	−1.45**	−0.69	−1.13, −0.34	32.17%
Escape acceptance	2.17***	−0.39***	−1.29**	−0.84	−1.28, −0.53	39.54%
**Preoccupied type (n = 101): RAAS avoidance**						
**JSE-HP total score**						
Neutral acceptance	−1.04*	1.47*	−3.83	−1.52	−3.95, −0.20	–
**Compassionate care**						
Neutral acceptance	−1.04*	0.52*	−2.81**	−0.54	−1.55, −0.01	16.06%
**Fearful type (*n* = 54): RAAS avoidance**						
**Compassionate care**						
Fear of death	4.36**	−0.58**	−2.28	−2.54	−6.84, −0.36	–

*p < 0.05;**p < 0.01;***p < 0.001.

In the preoccupied type, neutral acceptance (−) played a fully mediating role in the association of RAAS avoidance with JSE-HP total score (*p*s < 0.05) and a partial mediating role in the relationship between RAAS avoidance and compassionate care (*p*s < 0.05).

In the fearful type, fear of death played a fully mediating role in the relationship between RAAS avoidance and compassionate care (*p*s < 0.01).

**Figure 2:**
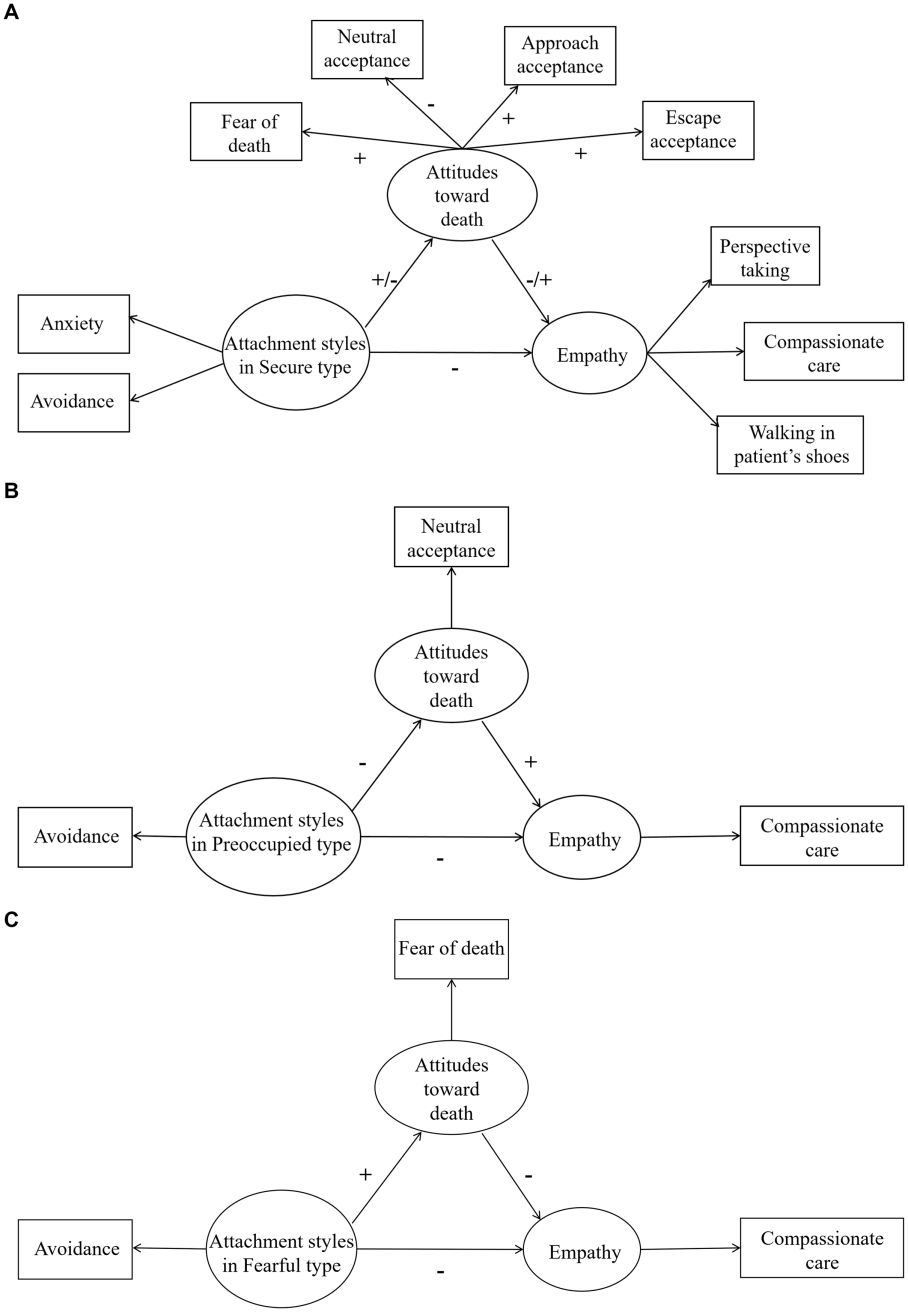
Schematic diagrams of mediation models for trainee nurses with **(A)** Secure attachment type, **(B)** Preoccupied type, and **(C)** Fearful type.

In the dismissing type, no mediating effect of individual attitude toward death was found (also see Figure 2).

### 5.4 Moderating role of death attitudes on the associations between attachment styles and empathy among trainee nurses with different attachment types

**Figure 3:**
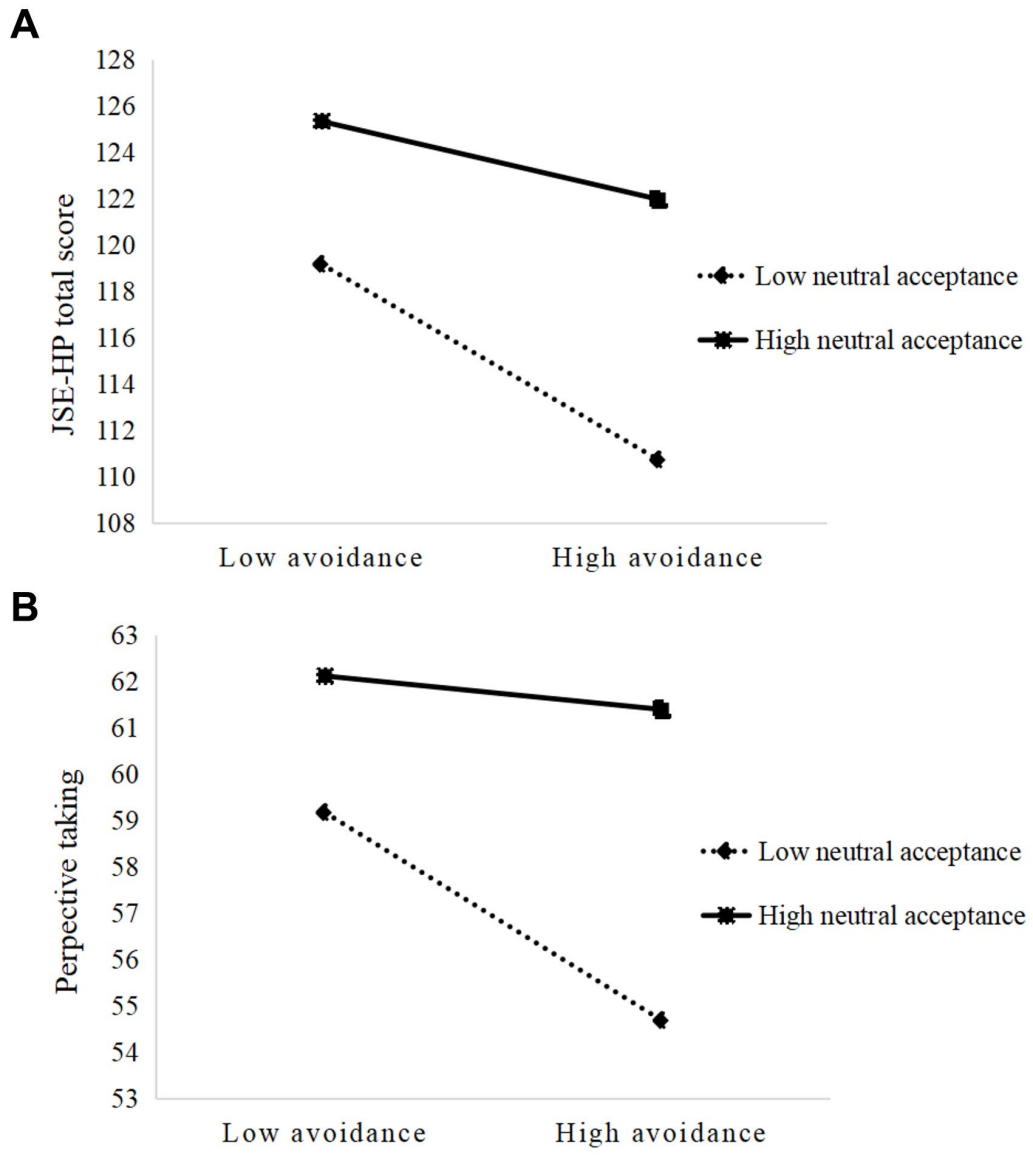
Interaction effect of neutral acceptance of death and attachment-related avoidance on **(A)** JSE-HP total score and **(B)** perspective taking in trainee nurses with secure attachment type.

As shown in Table 5, in the secure type, RAAS avoidance and its interaction with DAP-R, neutral acceptance negatively predicted JSE-HP total score (avoidance: *p* < 0.001; interaction: *p* < 0.05) and perspective taking (avoidance: *p* < 0.001; interaction: *p* < 0.01). These results indicated that neutral acceptance weakened the negative predictions of RAAS avoidance on JSE-HP total score and perspective taking in secure-type trainee nurses, as presented in Figure 3. No other moderating effect was found among trainee nurses with different attachment types.

**Table 5:** Moderating role of attitudes toward death on the associations between attachment styles and empathy levels in trainee nurses with different attachment types (*N* = 566).

Adjusting variable	β	SE	95% CI	*R* ^2^
**Secure type (*n* = 396)**				
**JSE-HP total score**				0.21
RAAS avoidance	−5.31***	0.99	−7.25, −3.37	
RAAS avoidance × neutral acceptance	0.84*	0.35	0.14, 1.53	
**Perspective taking**				0.43
RAAS avoidance	−2.34***	0.57	−3.45, −1.23	
RAAS avoidance × neutral acceptance	0.62**	0.20	0.22, 1.02	

*p < 0.05; **p < 0.01; ***p < 0.001.

## 6 Discussion

In this study, we explored the precise interrelationships between attachment styles, attitudes toward death, and empathy levels among Chinese trainee nurses with four attachment types, namely, the secure type, the preoccupied type, the dismissing type, and the fearful type. In general, the study found that there were differences in attitudes toward death and empathy levels among trainee nurses with these different attachment types. Moreover, attitudes toward death demonstrated mediating and moderating effects on the relationships between attachment styles and empathy, especially in the secure type. These results validated all our hypotheses.

More specifically, we found that in secure-type trainee nurses, RAAS avoidance and anxiety were positively correlated with DAP-R fear of death, death avoidance, escape acceptance, and approach acceptance and negatively correlated with neutral acceptance, JSE-HP total score, and all its subscale scores. These results suggested that these nurses, with less avoidant and anxious attachment tendencies (Collins, 1996), had less attitudes of fear of death or desire for death (Wong et al., 1994). However, they were more able to understand the positive aspects of death in life, to face death rationally and objectively (Schaufel et al., 2011), and exhibited higher empathic competence, including aspects of cognitive empathy, emotional empathy, and expression of empathy. A survey of nurses in five healthcare institutions in Lisbon also found a positive relationship between intimate attachment and neutral acceptance of death and a negative association of intimate attachment with anxious attachment; and dependent attachment was negatively associated and anxious attachment was positively associated with higher fear of death (Gama et al., 2012), which supported our findings. There was also a wealth of literature showing the impact of attachment on empathy. For example, a cross-sectional study of undergraduate nursing students in Australia found that more pronounced secure attachment was associated with higher levels of empathy (Williams et al., 2017). We also found that the JSE-HP total scores of securely attached individuals were positively correlated with neutral acceptance and negatively correlated with the other four dimensions of the DAP-R. In other words, the more rationally and objectively they view death and the less they fear or expect death, the higher their ability to empathize. These findings were supported by previous findings that through education related to death and dying, students had less fear of death, while their empathy levels were increased (Manolakis et al., 2011).

Based on previous studies, we further found that attitudes toward death mediated the relationships between attachment styles and empathy levels of securely attached nurses. Specifically, DAP-R fear of death, escape acceptance, approach acceptance, and neutral acceptance (−) partially mediated the associations between RAAS avoidance/anxiety and JSE-HP total score, as well as all its subscales scores, except that escape acceptance fully mediated the relationship between RAAS anxiety and perspective taking. These results indicated that a secure attachment style may increase all aspects of empathic performance including cognitive empathy, emotional empathy, and expression of empathy of trainee nurses by reducing their fear of death and their tendency to use death as a method of coping with real-world problems and increasing their rational attitude toward death. Meanwhile, less attachment anxiety leads to fewer thoughts of death as an escape from suffering, so they could have higher cognitive empathy with the patient. The results could be explained by the secure attachment style that has a buffering effect on fear of death (Maxfield et al., 2014; Mikulincer, 2019; Yetzer and Pyszczynski, 2019); and the more positive the attitude toward death, the higher the level of empathy overall (Han et al., 2023). On the other hand, the literature showed that acute stress hindered cognitive empathy (Nitschke and Bartz, 2023), whereas secure attachment individuals had better emotion regulation, which favored cognitive empathy (Henschel et al., 2020; Kämpf et al., 2023). In addition, we found that neutral acceptance negatively moderated the prediction of RAAS avoidance on JSE-HP total score and perspective taking of secure-type nurses, suggesting that rational attitudes toward death can attenuate the negative effect of avoidant attachment tendency on their ability to empathize, especially cognitive empathy. On the one hand, previous evidence indicated that avoidant attachment did have a negative effect on overall empathy level and cognitive empathy (Khodabakhsh, 2012; Kural and Kovács, 2022); on the other hand, Manolakis et al. (2011) found that module education on death and dying could lead to less fear of death and increased empathy levels among pharmacy students, all of which supported the moderation effect found in this study.

Among preoccupied type trainee nurses, first, we found that scores on fear of death, death avoidance, escape acceptance, and approach acceptance were higher than those of secure type nurses, and fear of death score was higher than that of the score of dismissing type, consistent with evidence that high anxious attachment style was associated with negative attitudes toward death such as fear of death, death avoidance, and escape acceptance (Choreva, 2020). Among cancer patients, preoccupied attachment type was also found to predict higher death anxiety (Scheffold et al., 2018). In terms of empathy, we found that nurses with preoccupied type had no significant deficits in cognitive empathy and emotional empathy, while they were less able to provide compassionate care, that is, expressing care, concern, and sympathy. As previous research studies have reported, anxious-ambivalent attached individuals did not always lack empathy (De Sanctis and Mesurado, 2022), but their positive other schema and negative self-schema, in which they perceive others as good but themselves as unlovable and unworthy of love (Hawkins et al., 2007), could prevent them from expressing the empathy they already possessed. Our further mediation analyses showed that neutral acceptance (−) fully mediated the negative relationship between RAAS avoidance and their JSE-HP total scores and partially mediated the negative relationship between RAAS avoidance and compassionate care, that is, less avoidant attachment styles could increase their overall empathic capacity by allowing for greater neutrality toward death but only partially increased their expression of empathy. When the avoidant attachment style was reduced, the fear of death was also reduced (Mikulincer, 2019). The nursing students were then able to bet on perceiving and understanding the suffering of others and develop a stronger motivation to alleviate their suffering (Han et al., 2023).

Similar to the preoccupied type trainee nurses, the fearful type nurses also had more negative attitudes toward death than the secure type nurses. In addition to similar high attachment-related anxiety as preoccupied type individuals (Collins, 1996), fearful type individuals had difficulties in emotion regulation (Ozeren, 2022), which may make it more difficult for them to regulate fear of death and dying when confronted with patients, or they may be more inclined to escape a troubled and painful life through death (Duran and Polat, 2024). In terms of empathy, our results showed that these nurses scored lower on the JSE-HP total score and all its subscale scores, suggesting that they had deficits in empathy, manifested widely by their inability to cognitively understand, emotionally empathize with, and effectively express empathy toward the patients. This may be related to their perception that they had limited interpersonal emotion regulation skills (George-Levi et al., 2022). In addition, their characteristic negative schemas of self and others could create a need to distance themselves from others and even use aggression to avoid intimacy (Péloquin et al., 2011; Cricchio et al., 2022). We also found that fear of death fully mediated the relationship between RAAS avoidance and compassionate care, implying that an avoidant attachment style led them to fear death and, therefore, fail to express care for patients. On the one hand, the avoidant style contributed to fear of death (Mikulincer, 2019); on the other hand, it could lead to poor self-concept clarity among those with attachment avoidance and anxiety (namely fearful type nurses), and self-concept clarity is important in interfering with personal distress and promoting empathic concern (Kural and Kovács, 2022).

Among dismissing type nurses, RAAS anxiety was positively correlated with death avoidance; JSE-HP total score and compassionate care were positively correlated with death avoidance and neutral acceptance. These findings suggested that as their anxiety in attachment relationships decreased (Collins, 1996), so did their death avoidance and empathic capacity, particularly expression of empathy. On the one hand, the literature showed that insecure attachment may increase the risk of suicidal thoughts and behaviors in individuals (Green et al., 2020), and on the other hand, individuals with dismissing type themselves tend to regulate their emotions by suppressing and denying their personal feelings (Mikulincer and Shaver, 2019), and such decreased self-compassion may also decrease their ability to empathize with others (Fuochi et al., 2018).

Furthermore, the influence of culture should be taken into account when developing empathy training programs as culture is fundamental in shaping empathic behavior (Yaghoubi Jami et al., 2024). In collectivist cultural norms, the overall degree of empathy and emotional empathy is higher (Chopik et al., 2016), whereas, in individualist cultural norms, higher self-orientation could lead to lower motivation to consider the needs of others and take their positions (Yaghoubi Jami et al., 2024). On the other hand, scholars reported a shared sense of empathy and awareness of the impact of death-related events, such as war, on humanity that transcends national boundaries (Yaghoubi Jami and Tabrizi, 2023). Such evidence provides more hints for empathy training related to the topic of pain and death in clinical settings across cultures.

This study also has some limitations. First, it was a cross-sectional design, and the relationships between attachment styles, attitudes to death, and empathy were inferred and needed to be validated by subsequent longitudinal studies. Second, although the current sample included junior nurses of different ages, genders, and educational statuses, it is worth noting that they were all from the same collectivist cultural norm, and the high degree of cultural homogeneity limited the external validity of the findings on empathy. Therefore, a subsequent large-scale, multicenter, cross-cultural validation of the findings was recommended. Third, the proportion of dismissing type nurses was quite low among the four attachment types, and the amount of data collected on this type was also small, thus further research is needed to validate this part of the results. Fourth, some sociodemographic information on the participants was not collected, such as their socioeconomic status and qualification of profession, which should be considered in future studies. Finally, it is recommended that observer-reported empathic behavior be recorded concurrently as there was evidence of differences between observer-reported and self-reported empathy in medical students (Chen et al., 2010).

## 7 Conclusion

The secure attachment style could increase cognitive empathy, emotional empathy, and expression of empathy in trainee nurses by reducing their fear or avoidance of death or by increasing their neutrality toward death; in contrast, the avoidant attachment style would reduce empathy, and in particular the expression of empathy, in insecurely attached nurses by increasing their fear of death. These findings suggest that appropriate education related to death should also be provided to trainee nurses with different attachment types, based on cultural commonalities and culturally specific empathy training, particularly to reduce fear of death in insecurely attached ones, in order to reduce the risk of empathy fatigue and improve quality of care.

## Data availability statement

The original contributions presented in the study are included in the article/supplementary material, further inquiries can be directed to the corresponding author.

## Ethics statement

The studies involving humans were approved by the Ethics Committee of Hangzhou Normal University School of Nursing (No. 2024029). The studies were conducted in accordance with the local legislation and institutional requirements. The participants provided their written informed consent to participate in this study.

## Author contributions

TW: Writing – original draft, Writing – review & editing. MG: Formal analysis, Validation, Writing – review & editing. HJ: Conceptualization, Resources, Writing – review & editing. BZ: Writing – review & editing, Conceptualization, Data curation, Funding acquisition, Methodology, Project administration, Writing – original draft.

## References

[ref1] Amir K. Okalo P. (2022). Frontline nurses’ compassion fatigue and associated predictive factors during the second wave of COVID-19 in Kampala, Uganda. Nurs. Open 9, 2390–2396. doi: 10.1002/nop2.1253, PMID: 35633514 PMC9348371

[ref2] Chachula K. M. (2022). A comprehensive review of compassion fatigue in pre-licensure health students: antecedents, attributes, and consequences. Curr. Psychol. 41, 6275–6287. doi: 10.1007/s12144-020-01122-3 33078054 PMC7558253

[ref3] Chen D. C. Pahilan M. E. Orlander J. D. (2010). Comparing a self-administered measure of empathy with observed behavior among medical students. J. Gen. Intern. Med. 25, 200–202. doi: 10.1007/s11606-009-1193-4 20013070 PMC2839329

[ref4] Chopik W. J. O’Brien E. Konrath S. H. (2016). Differences in empathic concern and perspective taking across 63 countries. J. Cross-Cult. Psychol. 48, 23–38. doi: 10.1177/0022022116673910

[ref5] Choreva K. (2020). An investigation of the relationship between adult attachment styles and attitudes toward death and dying. (Doctoral Dissertation, Alliant International University).

[ref6] Collins N. L. (1996). Working models of attachment: implications for explanation, emotion, and behavior. J. Pers. Soc. Psychol. 71, 810–832. doi: 10.1037/0022-3514.71.4.810, PMID: 8888604

[ref7] Coulehan J. L. Platt F. W. Egener B. Frankel R. Lin C. T. Lown B.. (2001). Let me see if i have this right…: words that help build empathy. Ann. Intern. Med. 135, 221–227. doi: 10.7326/0003-4819-135-3-200108070-00022, PMID: 11487497

[ref8] Cricchio M. G. L. Musso P. Coco A. L. Cassibba R. Liga F. (2022). The relation between empathy and aggression: the role of attachment style. Eur. J. Psychol. 18, 319–336. doi: 10.5964/ejop.4509, PMID: 36348816 PMC9632554

[ref9] Cuff B. M. Brown S. J. Taylor L. Howat D. J. (2016). Empathy: a review of the concept. Emot. Rev. 8, 144–153. doi: 10.1177/1754073914558466

[ref10] Damiano R. F. de Andrade Ribeiro L. M. Dos Santos A. G. Da Silva B. A. Lucchetti G. (2017). Empathy is associated with meaning of life and mental health treatment but not religiosity among Brazilian medical students. J. Relig. Health 56, 1003–1017. doi: 10.1007/s10943-016-0321-9, PMID: 27783262

[ref11] De Sanctis F. Mesurado B. (2022). Attachment style and empathy in late children, adolescents, and adults: meta-analytic review. Int. J. Psychol. Res. 15, 114–129. doi: 10.21500/20112084.5409, PMID: 37274515 PMC10233954

[ref12] Decety J. (2020). Empathy in medicine: what it is, and how much we really need it. Am. J. Med. 133, 561–566. doi: 10.1016/j.amjmed.2019.12.012 31954114

[ref13] Duran S. Polat S. (2024). Nurses’ attitudes towards death and its relationship with anxiety levels. OMEGA J. Death Dying 88, 1530–1544. doi: 10.1177/00302228211065963, PMID: 34982589

[ref14] Ein-Dor T. Hirschberger G. (2016). Rethinking attachment theory: from a theory of relationships to a theory of individual and group survival. Curr. Dir. Psychol. Sci. 25, 223–227. doi: 10.1177/0963721416650684

[ref15] Fuochi G. Veneziani C. A. Voci A. (2018). Exploring the social side of self-compassion: relations with empathy and outgroup attitudes. Eur. J. Soc. Psychol. 48, 769–783. doi: 10.1002/ejsp.2378

[ref16] Gama G. Vieira M. Barbosa F. (2012). Factors influencing nurses’ attitudes toward death. Int. J. Palliat. Nurs. 18, 267–273. doi: 10.12968/ijpn.2012.18.6.267 22885899

[ref17] Garnett A. Hui L. Oleynikov C. Boamah S. (2023). Compassion fatigue in healthcare providers: a scoping review. BMC Health Serv. Res. 23:1336. doi: 10.1186/s12913-023-10356-3, PMID: 38041097 PMC10693134

[ref18] George-Levi S. Laslo-Roth R. Schmidt-Barad T. (2022). Feeling you, when you feel me: attachment, empathic concern, and interpersonal emotion regulation. J. Soc. Psychol. 162, 655–669. doi: 10.1080/00224545.2021.1940075, PMID: 34315350

[ref19] Graves J. Joyce C. Hegazi I. (2023). “From empathy to compassion fatigue: A narrative review of implications in healthcare” in Empathy: Advanced Research and Applications, 1–28.

[ref20] Green J. Berry K. Danquah A. Pratt D. (2020). The role of psychological and social factors in the relationship between attachment and suicide: a systematic review. Clin. Psychol. Psychother. 27, 463–488. doi: 10.1002/cpp.2445, PMID: 32167194

[ref21] Han S. R. Li Q. F. Lian S. L. Zhang Z. X. Liu L. M. Sun C. Q. (2023). Mediating effects of empathy between nursing students’ personality traits and attitudes toward death. Chinese J. Behav. Med. Brain Sci. 32, 442–447. doi: 10.3760/cma.j.cn371468-20221019-00620

[ref22] Harrison K. (2021). Compassion fatigue: understanding empathy. Vet. Clin. Small Anim. Pract. 51, 1041–1051. doi: 10.1016/j.cvsm.2021.04.020, PMID: 34218949

[ref23] Hawkins A. C. Howard R. A. Oyebode J. R. (2007). Stress and coping in hospice nursing staff. The impact of attachment styles. Psycho-oncology: journal of the psychological, social and behavioral dimensions of. Cancer 16, 563–572. doi: 10.1002/pon.1064 17004295

[ref24] Hayes A. F. (2017). Introduction to mediation, moderation, and conditional process analysis: a regression-based approach. (2nd ed.). New York: Guilford Publications. 51 335–337.

[ref25] Henschel S. Nandrino J. L. Doba K. (2020). Emotion regulation and empathic abilities in young adults: the role of attachment styles. Personal. Individ. Differ. 156:109763. doi: 10.1016/j.paid.2019.109763

[ref26] Hinde R. A. (1969). John Bowlby, attachment and loss. I. Attachment. London: Hogarth Press 63s.793.

[ref2002] Hirsch E. M. (2007). The role of empathy in medicine: a medical student’s perspective. AMA Journal of Ethics. 9, 423–427. 10.1001/virtualmentor.2007.9.6.medu1-0706 23218048

[ref27] Hojat M. DeSantis J. Shannon S. C. Mortensen L. H. Speicher M. R. Bragan L.. (2018). The Jefferson scale of empathy: a nationwide study of measurement properties, underlying components, latent variable structure, and national norms in medical students. Adv. Health Sci. Educ. 23, 899–920. doi: 10.1007/s10459-018-9839-9, PMID: 29968006 PMC6245107

[ref28] Hojat M. Gonnella J. S. Nasca T. J. Mangione S. Vergare M. Magee M. (2002). Physician empathy: definition, components, measurement, and relationship to gender and specialty. Am. J. Psychiatry 159, 1563–1569. doi: 10.1176/appi.ajp.159.9.1563, PMID: 12202278

[ref29] Hu M. Zhang Z. Ou Y. Zhang H. Zheng X. Wu Y.. (2023). Importance of the nurses’ empathy level in operating rooms. Altern. Ther. Health Med. 29, 107–111. 37023311

[ref30] Huh H. J. Kim K. H. Lee H. K. Chae J. H. (2017). Attachment styles, grief responses, and the moderating role of coping strategies in parents bereaved by the Sewol ferry accident. Eur. J. Psychotraumatol. 8:1424446. doi: 10.1080/20008198.2018.1424446, PMID: 29372009 PMC5774413

[ref31] Kämpf M. S. Adam L. Rohr M. K. Exner C. Wieck C. (2023). A meta-analysis of the relationship between emotion regulation and social affect and cognition. Clin. Psychol. Sci. 11, 1159–1189. doi: 10.1177/21677026221149953

[ref32] Kazmierczak M. (2015). Couple empathy–the mediator of attachment styles for partners adjusting to parenthood. J. Reprod. Infant Psychol. 33, 15–27. doi: 10.1080/02646838.2014.974148

[ref33] Khodabakhsh M. (2012). Attachment styles as predictors of empathy in nursing students. J. Med. Ethics Hist. Med. 5:8. 23908761 PMC3715012

[ref34] Kural A. I. Kovács M. (2022). The association between attachment orientations and empathy: the mediation effect of self-concept clarity. Acta Psychol. 229:103695. doi: 10.1016/j.actpsy.2022.103695, PMID: 35930953

[ref35] Manolakis M. L. Olin J. L. Thornton P. L. Dolder C. R. Hanrahan C. (2011). A module on death and dying to develop empathy in student pharmacists. Am. J. Pharm. Educ. 75:71. doi: 10.5688/ajpe75471, PMID: 21769147 PMC3138342

[ref36] Maxfield M. John S. Pyszczynski T. (2014). A terror management perspective on the role of death-related anxiety in psychological dysfunction. Humanist. Psychol. 42, 35–53. doi: 10.1080/08873267.2012.732155

[ref37] McKenna L. Boyle M. Brown T. Williams B. Molloy A. Lewis B.. (2012). Levels of empathy in undergraduate nursing students. Int. J. Nurs. Pract. 18, 246–251. doi: 10.1111/j.1440-172X.2012.02035.x 22621294

[ref38] Medina-Fernández J. Torres-Soto N. Y. Casco-Gallardo K. Ruiz-Lara A. Martínez-Ramírez B. Fuentes-Fernández E. (2023). Fear and cope with death in intensive care nurses: a structural model predictor of compassion fatigue. Invest. Educ. Enferm. 41:e12. doi: 10.17533/udea.iee.v41n1e12, PMID: 37071867 PMC10152909

[ref39] Melchers M. C. Li M. Haas B. W. Reuter M. Bischoff L. Montag C. (2016). Similar personality patterns are associated with empathy in four different countries. Front. Psychol. 7:173343. doi: 10.3389/fpsyg.2016.00290 PMC4781828 27014115

[ref40] Mikulincer M. (2019). “An attachment perspective on managing death concerns” in Handbook of terror management theory (Amsterdam: Academic Press), 243–257.

[ref41] Mikulincer M. Shaver P. R. (2019). Attachment orientations and emotion regulation. Curr. Opin. Psychol. 25, 6–10. doi: 10.1016/j.copsyc.2018.02.006 29494853

[ref42] Nitschke J. P. Bartz J. A. (2023). The association between acute stress & empathy: a systematic literature review. Neurosci. Biobehav. Rev. 144:105003. doi: 10.1016/j.neubiorev.2022.105003 36535374

[ref43] Ozeren G. S. (2022). The correlation between emotion regulation and attachment styles in undergraduates. Perspect. Psychiatr. Care 58, 482–490. doi: 10.1111/ppc.12902, PMID: 34223633

[ref44] Péloquin K. Lafontaine M. F. Brassard A. (2011). A dyadic approach to the study of romantic attachment, dyadic empathy, and psychological partner aggression. J. Soc. Pers. Relat. 28, 915–942. doi: 10.1177/0265407510397988

[ref45] Peng Y. Zhao L. Shen M. D. Ling L. Zou H. J. (2018). Analysis of undergraduate nursing students’ attitudes toward death and their influencing factors. Nurs. Res. 32, 380–383. doi: 10.3969/j.issn.1009-6493.2018.03.013

[ref46] Pérez-Chacón M. Chacón A. Borda-Mas M. Avargues-Navarro M. L. (2021). Sensory processing sensitivity and compassion satisfaction as risk/protective factors from burnout and compassion fatigue in healthcare and education professionals. Int. J. Environ. Res. Public Health 18:611. doi: 10.3390/ijerph18020611, PMID: 33445789 PMC7828252

[ref47] Russ V. Stopa L. Sivyer K. Hazeldine J. Maguire T. (2022). The relationship between adult attachment and complicated grief: a systematic review. OMEGA J. Death Dying:302228221083110. doi: 10.1177/00302228221083110 PMC11423550 35635029

[ref48] Schaufel A. M. Nordrehaug J. E. Malterud K. (2011). Hope in action—facing cardiac death: a qualitative study of patients with life-threatening disease. Int. J. Qual. Stud. Health Well Being 6:5917. doi: 10.3402/qhw.v6i1.5917, PMID: 21423599 PMC3061819

[ref49] Scheffold K. Philipp R. Koranyi S. Engelmann D. Schulz-Kindermann F. Härter M.. (2018). Insecure attachment predicts depression and death anxiety in advanced cancer patients. Palliat. Support. Care 16, 308–316. doi: 10.1017/S1478951517000281, PMID: 28502270

[ref50] Servaty H. L. Krejci M. J. Hayslip B. Jr. (1996). Relationships among death anxiety, communication apprehension with the dying, and empathy in those seeking occupations as nurses and physicians. Death Stud. 20, 149–161. doi: 10.1080/07481189608252747, PMID: 10178138

[ref51] Sinclair S. Raffin-Bouchal S. Venturato L. Mijovic-Kondejewski J. Smith-MacDonald L. (2017). Compassion fatigue: a meta-narrative review of the healthcare literature. Int. J. Nurs. Stud. 69, 9–24. doi: 10.1016/j.ijnurstu.2017.01.003 28119163

[ref52] Tang L. Zhang L. Li Y. Zhou L. Cui J. Meng X.. (2014). Validation and reliability of a Chinese version death attitude profile-revised (DAP-R) for nurses. J. Nurs. Sci. 29, 64–66. doi: 10.3870/hlxzz.2014.14.064

[ref53] Walker L. O. Avant K. C. (2005). Strategies for theory construction in nursing. Upper Saddle River, NJ: Pearson Prentice Hall.

[ref54] Ward J. Cody J. Schaal M. Hojat M. (2012). The empathy enigma: an empirical study of decline in empathy among undergraduate nursing students. J. Prof. Nurs. 28, 34–40. doi: 10.1016/j.profnurs.2011.10.007, PMID: 22261603

[ref55] Wells-English D. (2019). Compassion fatigue and satisfaction: influence on turnover among oncology nurses at an urban cancer center. Clin. J. Oncol. Nurs. 23, 487–493. doi: 10.1188/19.CJON.487-493 31538984

[ref56] Wibisono S. Minto K. Lizzio-Wilson M. Thomas E. F. Crane M. Molenberghs P.. (2022). Attitudes toward and experience with assisted-death services and psychological implications for health practitioners: a narrative systematic review. OMEGA J. Death Dying:302228221138997. doi: 10.1177/00302228221138997 36357863

[ref57] Williams B. Brown T. McKenna L. Beovich B. Etherington J. (2017). Attachment and empathy in Australian undergraduate paramedic, nursing and occupational therapy students: a cross-sectional study. Collegian 24, 603–609. doi: 10.1016/j.colegn.2016.11.004

[ref58] Wong P. T. Reker G. T. Gesser G. (1994). The death attitude profile-revised: A multidimensional measure of attitudes toward death. Death anxiety handbook: Research, instrumentation, and application. Washington, D.C.: Taylor & Francis.

[ref59] Wu W. L. Zhang W. Liu X. H. (2004). The reliability and validity of adult attachment scale (AAS-1996 revised edition): a report on its application in China. Sichuan da xue xue bao. Yi xue ban= journal of Sichuan University. Med. Sci. Ed. 35, 536–538. doi: 10.3969/j.issn.1672-173X.2004.04.025 15291121

[ref60] Yaghoubi Jami P. Tabrizi K. (2023). Contemporary mirror imaging between American and Iranian citizens: an exploratory mixed-method research study. Psych 5, 724–741. doi: 10.3390/psych5030047

[ref61] Yaghoubi Jami P. Walker D. I. Mansouri B. (2024). Interaction of empathy and culture: a review. Curr. Psychol. 43, 2965–2980. doi: 10.1007/s12144-023-04422-6

[ref63] Yetzer A. M. Pyszczynski T. (2019). “Terror management theory and psychological disorder: ineffective anxiety-buffer functioning as a transdiagnostic vulnerability factor for psychopathology” in Handbook of terror management theory (Amsterdam: Academic Press), 417–447.

